# Push-out Bond Strength of Bulk-Fill Composites Used as Intracanal Anchorage Material

**DOI:** 10.3290/j.jad.c_1991

**Published:** 2025-04-30

**Authors:** Richard Sturm, Hagay Shemesh, Emely Bortel, Bernhard Hesse, Kerstin Bitter

**Affiliations:** a Richard Sturm Dentist, Department of Operative, Preventive and Pediatric Dentistry, Charité – Universitätsmedizin Berlin, Germany. Researched, designed and carried out the study, wrote the manuscript.; b Hagay Shemesh Associate Professor, Department of Endodontology, Academic Center of Dentistry Amsterdam, University of Amsterdam and VU University, Amsterdam, The Netherlands. Designed the study, carried out result interpretation, proofread the whole manuscript.; c Emely Bortel Researcher, XPLORAYTION, Bismarckstraße 10–12, 10625 Berlin, Germany. Carried out imaging and image analyses, wrote specific parts of M&M, proofread the whole manuscript.; d Bernhard Hesse Researcher, XPLORAYTION, Bismarckstraße 10–12, 10625 Berlin, Germany. Carried out imaging and image analyses, wrote specific parts of M&M, proofread the whole manuscrip.t; e Kerstin Bitter Professor, Department of Operative Dentistry and Periodontology, Medical Faculty, Martin-Luther University Halle-Wittenberg, Germany. Researched, designed and carried out the study, wrote the manuscript.

**Keywords:** 3D analysis, adhesive/material interface, bulk-fill composite, intracanal anchorage, micro-CT, post endodontic restoration, push-out bond strength, porosity

## Abstract

**Purpose:**

Evaluation of different composites with varying viscosity for their suitability as intracanal anchorage (ICA) materials using push-out bond strength testing.

**Materials and Methods:**

48 human maxillary incisors were root filled, crowns partially removed except one residual wall, and distributed into four groups (n = 12), according to one of the following ICA composites: Ormocer (AFx), preheated composite (VB), core build-up (RDC) or flowable (SDR). A 4 mm deep root canal enlargement was prepared using Gates Glidden burrs, and hard tissues were conditioned using a universal adhesive in etch-and-rinse mode. Intracanal cavities were filled using the groups’ specific ICA material and a nanohybrid composite (GrandioSO, VOCO) for crown reconstruction. Two samples per group were scanned using phase-contrast-enhanced µ-computer tomography (PCE-CT). The remaining samples were thermo-mechanically loaded (TML), and push-out bond strengths and fracture patterns of ICA materials were analyzed.

**Results:**

Push-out bond strengths were significantly affected by ICA materials (P = 0.001) and location inside the root canal (P < 0.005; generalized estimating equations). VB showed a significantly lower bond strength (13.5MPa ± 5.3MPa) compared to RDC (19.6MPa ±7.6MPa) and AFx (21.4MPa ±7.6MPa), but did not differ significantly from SDR (20MPa ± 10.3MPa). All groups demonstrated predominantly adhesive failures between the composite and dentin (P < 0.05; Chi-square test). µ-CT scans indicate material-dependent localization and quantity of voids.

**Conclusion:**

The survival rates after TML and the push-out bond strength values indicate a sufficient bonding of all ICA materials. Frequently occurring voids highlight problems of application and the effects of materials’ viscosity on void formation and bond strength.

The need for root canal treatment is frequently accompanied by substantial loss of coronal tooth structure caused by caries or dental trauma. With respect to the amount of coronal hard tissue loss, various restoration procedures and strategies have been recommended for root-canal-treated teeth.^34^ Teeth with a high loss of coronal tooth structure are frequently restored with a post and core build-up followed by a crown restoration. Until today, the literature recommends post-placement for teeth with two or less remaining coronal walls, especially for maxillary anterior teeth due to high shear forces.^53^ Nevertheless, post-placement often comes along with extra loss of tooth structure due to post space preparation and root canal transportation.^23^ This enlargement and transport of the root canal can even be the cause of root canal perforations.^29^ Also, a loss of inner cervical dentin can negatively affect the survival of root-canal-treated teeth.^3^ A recent review was not able to demonstrate a positive effect of post-placement *in vivo* ^39^ regardless of tooth type or coronal restoration. Based on this knowledge and the fact that the post space preparation is the treatment step that causes the second-highest loss of tooth structure after endodontic access preparation,^24^ hard-tissue-saving treatment options are needed.

It is, therefore, of great importance to test materials for their suitability as intracanal anchorage (ICA). Unfortunately, there are no studies testing intracanal composite fillings for their bonding performance inside the root canal of mature teeth. The push-out bond strength inside the root canal is mostly tested in combination with glass fiber posts. The performance of intracanal composite fillings is frequently tested for their maximum load capability in comparison to metal or glass fiber posts. *In-vitro *studies showed that intracanal composite fillings can achieve equal maximum load capabilities as glass fiber posts in mature premolars,^25^ but inside central incisors the intracanal filling showed significantly lower maximum load capabilities.^59^ An *in vivo* study revealed promising results for primary front teeth in need of a build-up of the complete crown, when intracanal composite fillings were placed for build-up anchorage. Success rates of 71% after 18 months for their 3- to 5-year-old patients were achieved.^17^

Bulk-fill composites are promising materials for intracanal anchorage, because they allow light curing to penetrate in thicker increments of composite and to greater depths.^31^ The material-specific polymerization shrinkage and viscosity of bulk-fill materials are determined by monomer composition and filler content.^5, 30^ In comparison to conventional composites, bulk-fill composites demonstrate a similar or reduced tendency to undergo shrinkage stress, particularly in small class I cavities,^48,65^ such as those commonly observed in root canal orifices.^60^

A study demonstrated that low shrinkage composites, when compared to conventional composites, did not exhibit any advantage based on the reduced shrinkage observed when the microtensile bond strength was evaluated in class I cavities (4 mm × 4 mm × 2.5 mm).^66^ However, it was discovered that the application mode has a considerable impact on the microtensile bond strength, with the highest results observed for lining with a flowable bulk-fill, followed by incremental application of further composite.^61^ In conclusion, the use of bulk-fill materials within the root canal orifice for adhesive retention appears to be an appropriate selection.

In order to assess the internal surfaces of the ICA, micro-CT imaging was conducted. This represented a significant improvement over previous methods, like sectioning^21^ or dye infiltration,^40^ which required further processing of the samples before analyzation. Since this processing of the samples harbors the risk of artificial damages, that possibly affect the results.^69^

Non-destructive sample evaluations reduce the risk of sample preparation artifacts and allow the analysis of the same samples multiple times, for example, after artificial aging. Micro-CT imaging can achieve a high spatial resolution^40^ and in dental research it can be helpful when underpinned with additional evaluations, for example, scanning electron microscopy, as shown by Sampaio et al.^50^ However, micro-CT image analyses pose a challenge when materials with similar radio-opacity are imaged.^7^ Although adding contrast agents improves the contrast, they can also interfere with the radiograph beams and cause radiographic artifacts as well as alter the materials’ properties.^40^ One effective solution to increase the contrast between different materials is synchrotron-based phase-contrast-enhanced micro-CT (PCE-CT).^14^ This method enables an easier differentiation of materials with similar radio-opacity and has earlier been used to analyze materials bonded into the root canal^58^ or in the orifice area.^26^

The aim of the current study was to characterize the adhesion of four composites, used as ICA, inside the root canal orifice. Three bulk-fill and one dual-curing build-up material, all with diverse viscosity, were used: a bulk-fill ormocer, a dual-curing build-up composite, a flowable bulk-fill composite, and a preheated bulk-fill composite (Table 1) were placed inside the root canal. Additionally, two exemplary samples of each material were controlled using high-resolution phase contrast-enhanced micro-computed tomography (PCE-CT). The null hypothesis of this study was that bond strengths inside the root canal orifice would not be affected by the composite material used, additional qualitative analyses of the interface between tooth substrate and composite aimed to analyze the effects of materials’ viscosity on the occurrence of voids.

**Table 1:** Materials used and group information

Material	Group shortcut	Material characteristic	Viscosity	Sample size (n)	Perimeter in mm^2^	LOT
Futurabond U		Dual-curing universal adhesive		1849378
GrandioSO	Nanohybrid composite	2044623
Admira Fusion xtra	AFx	bulk-fill, Ormocer	High	12	44,6	2045542
VisCalor Bulk	VB	Preheatedbulk-fill composite	Variable(temperature-dependent)	12	44,3	2046140
Rebilda DC	RDC	Dual-curing build-up composite	Low	12	44,4	1849515
SDR	SDR	flowable bulk-fill composite	Low	12	44,3	2168


## MATERIALS AND METHODS

### Sample Preparation

Forty-eight human maxillary incisors were selected from a tooth bank (approved by the Ethical Review Committee of the Charité – Universitätsmedizin Berlin EA4/102/14) and measured in mesiodistal and vestibulo-oral direction at the cementoenamel junction using a gauge to calculate the dimensions of all samples at the cervical level. All teeth had a minimum root length of 14 mm from the cementoenamel junction to the tip of the root and showed sound hard tissues. To achieve groups of homogenous diameters at the cervical level, the samples were lined up from big to small and then sorted in the following way 1, 2, 3, 4, 4, 3, 2, 1, 1, 2, … to four groups (G) (n = 12). Prior to the experiment, no sample size calculation was performed due to the limit of similar experimental set-ups. However, related recently published *in-vitro* studies also tested 12 samples per group.^10,13,33,56^ Impressions of the crowns of each tooth were made of Silaplast futur (Detax & Co. KG, Ettlingen, Germany). An overview of the experimental workflow is given in Figure 1. Class IV cavities were prepared using a diamond saw (sawblade 0.2 mm; EXAKT Advanced Technologies, Norderstedt, Germany), removing 2/3 of the incisal edge, stopping 1 mm above the cementoenamel junction (Fig 1a).

All samples were endodontically treated. Working length was determined 1 mm before the anatomical apex after initial scouting of the canal with a Flexicut file (Dentsply Sirona, Charlotte, USA) of ISO 10 and marked with a silicone stopper. Instrumentation was conducted using rotary ProTaperNext files up to size X5 (Ø/ taper 0.5 mm/ 6%; Dentsply Sirona), throughout instrumentation the canals were rinsed using 1% sodium hypochlorite (NaOCl) solution. Final irrigation was performed using NaOCl 1% followed by irrigation using 17% ethylenediaminetetraacetic acid (EDTA), both with ultrasonic activation (ultrasonic insert 25/25 IRRI S, 28-36 kHz oscillation frequency; VDW Endo Ultrasonic, VDW, Munich, Germany).^9^ After drying the canals using paper points, the root canal filling was placed using gutta-percha and an epoxy resin-based sealer (AH plus; Dentsply Sirona) in warm vertical compaction, removing the first 4 mm of gutta-percha at the canal orifice for filling placement. The coronal part of the root canal was enlarged using a Gates Glidden bur size 6 to ensure a minimum cavity size of 1.5 mm diameter for the intracanal composite anchorage to a depth of 4 mm. All edges of the class IV cavity located in enamel were beveled over 1 mm width in a 45° angle.

Table 1 summarizes the general information about restoration materials, such as material characteristics, the mean perimeter of the samples at the height of the cementoenamel junction per group, and the LOT numbers of the materials.

Subsequently, the cavity was thoroughly cleaned with alcohol and rinsed using water. Root canal dentin and coronal dentin were etched for 15 s and enamel for 30 s using 35% phosphoric acid (Vococid; VOCO, Cuxhaven, Germany) and again rinsed for 30 s with water. Afterwards, a multi-mode adhesive system (Futurabond U, VOCO) was applied for 30 s using a microbrush, air dried, and light cured (time 30s, wavelength range 385–515, intensity 1000 mW/cm^2^; Valo, Ultradent, South Jordan, UT, USA). Composites for ICA (Table 1) were used according to the manufacturer’s instructions.

The flowable bulk-fill SDR (Dentsply Sirona) was injected placing the application tip onto the gutta-percha until the complete cavity was filled. Rebilda DC (RDC; VOCO), the second low-viscosity material, was applied the same way as SDR (Dentsply Sirona). The high viscosity bulk-fill ormocer Admira Fusion xtra (AFx; VOCO) was placed directly from the capsular onto a plugger and was condensed inside the cavity. The fourth material used, VisCalor bulk (VB; VOCO), is a bulk-fill material that comes with a special heating dispenser to reduce the viscosity nearly to that of a flowable composite. It was injected in two steps and condensed as AFx. Coronal class IV restorations were made using a nanohybrid composite (GrandioSO; VOCO) with the help of impressions of the original crowns to reconstruct the original shape (Fig 1b). The composite was placed in 2 mm thick increments and after every increment, the material was light cured (time 20 s, wavelength range 385–515, intensity 1000 mW/cm^2^; Valo) whereby the device was placed as near as possible to the filling’s material.

### CT Imaging

**Fig 2:**
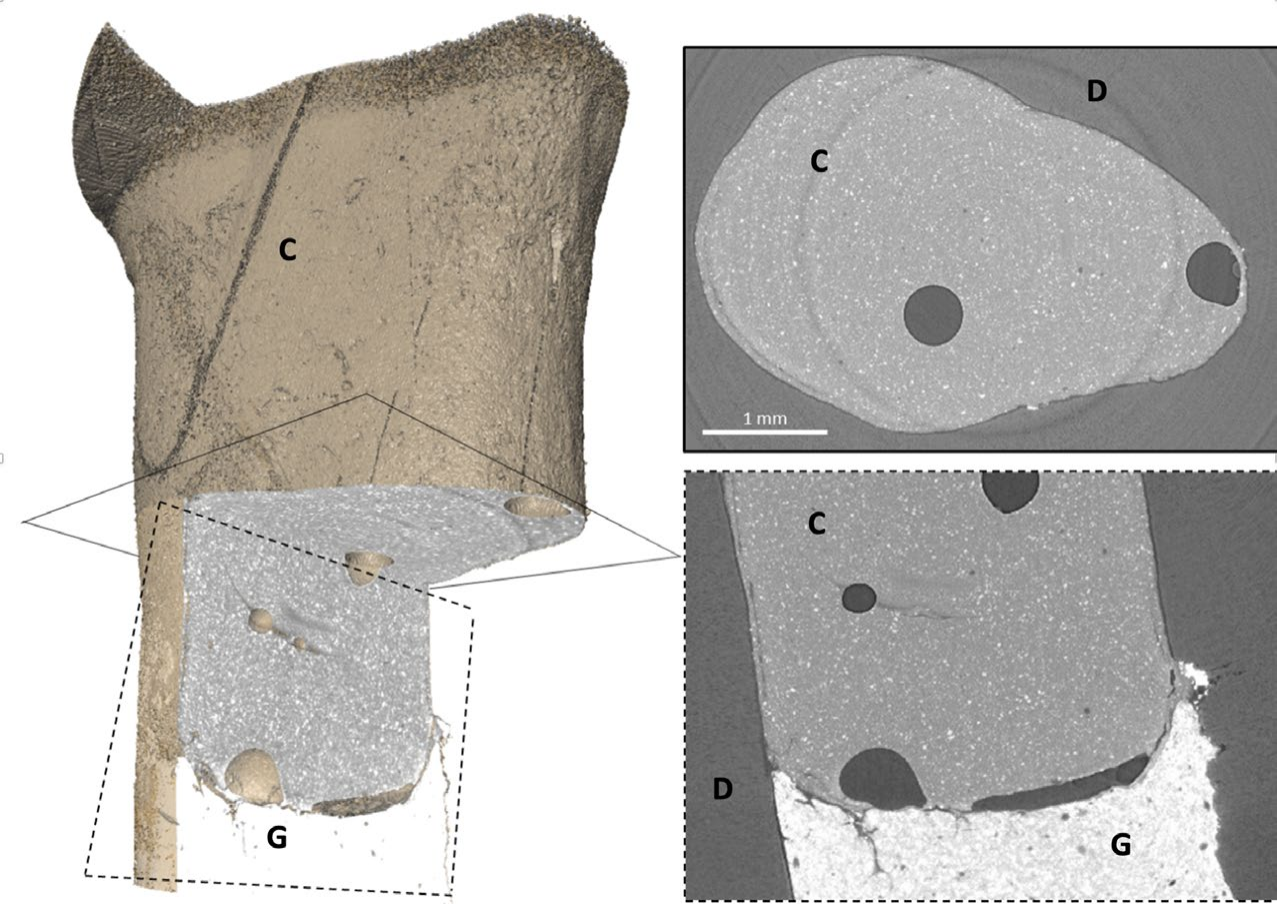
Exemplary representation of PCE-CT data obtained for an ICA sample. In the 3D cut-open rendering on the left side, the dentin is set to full transparency to see the ICA and the adjacent gutta-percha. The corresponding apical (continuous line) and lateral (dashed line) cross-sections are shown in the right panel. The phase-contrast enhancement allows clear identification of ICA material, surrounding dentin, gutta-percha, and pores. C = Composite (ICA), D = Dentin, G = Gutta-percha.

After sample preparation, two samples of each group were fixated inside a plastic tube for the µCT scans. To avoid drying out the samples, a moist sponge was added to the lid of the tube. The samples were scanned using synchrotron PCE-CT (Anatomix Beamline, Synchrotron Soleil, France^51^), from the incisal edge to a point 1 mm below the ICA inside the root canal. Scanning parameters were set at 40 keV for the X-ray energy and an isotropic voxel size of 3.07 µm (Fig 1c). In each scan, ~3900 radiographs were recorded over a 360° rotation using a Hamamatsu Orca Flash 4.0 V2 camera with an acquisition time of 100 ms per radiograph. Paganin‘s method^46^ combined with filtered back projection were applied for phase retrieval and tomographic reconstruction. Both calculation steps were performed simultaneously using in-house codes of the Anatomix beamline relying on PyHST, a software developed at the ESRF.^37^ For each sample, minimum intensity projections along 800 slices are calculated. Therewith, the information contained in the 3D dataset is projected along the composite filling‘s long axis onto one single slice, displaying the lowest gray value (pores) along the path of projections. Thus, a qualitative impression of the pores (size and distribution) contained within the post is achieved (Fig 2). For quantification of the pores, the datasets were binarized by applying an automatic histogram-based threshold based on Otsu’s algorithm, individually calculated for each dataset.^44^ The segmented shaft mask surface was morphologically filtered (cleaned) by an opening step with a spherical structuring element of 3. The pores were then extracted by a fill-hole step (implemented in ImageJ).^52^ Each pore bigger than 33 voxels was assigned an index by a connected component labeling approach. The pores volume was then examined via a pore-size histogram. To determine the spatial distribution of the pores, a distance map was calculated on the post mask, assigning to each voxel its shortest distance to the closest surface. By multiplying this distance map with the pore mask, for each pore its mean distance to the dentin surface was computed.

### Thermomechanical Loading

The remaining 10 samples per group were embedded for thermomechanical loading using a polyether impression material (Impregum, 3M Espe, Landsberg am Lech, Germany) and Technovit 4071 (Kulzer, Hanau, Germany). The samples were loaded with 50 N 1.2 million times at a 45° angle, with simultaneous thermal stressing (TML) from 5° to 55°C for 10,000 times (SD Mechatronik, Feldkirchen-Westerham, Germany).

### Push-out Testing

Subsequently, the samples were decoronated 1 mm above the cementoenamel junction and ground to the proximal filling margin perpendicular to the long axis of the root. Starting from this point, the samples were sectioned in 1 mm thick slices perpendicular to the long axis of the root using a band saw (sawblade 0.2 mm; EXAKT) until the root-canal-filling material occurred, resulting in two to three slices per sample: a coronal, a middle and an apical one (Fig 1c). The slices were controlled under 2.5× magnification (stereomicroscope Technival 2; Carl Zeiss, Jena, Germany) to ensure no pretest damages like dislodgment of the filling occurred. The bond strength of the intracanal composite filling material was tested using push-out tests, with a forehead speed of 0.5 mm/min in the cervical direction, using a universal testing machine and matching metal plungers (Zwick Roell; Ulm, Germany) (Fig 1d). The metal plunger was slightly smaller than the filling surface, so the plunger had flat contact with the composite but did not touch the dentin. For evaluation of the push-out bond strength, the apical slides were left out due to the low number of samples.

### Failure Analysis

Failures of the intracanal fillings were analyzed using an optical multifocus microscope (Fig 1e) (Keyence Corporation, Osaka, Japan) at a 30° angle and divided into (1) adhesive failures between composite and dentin, (2) cohesive failures inside the dentin or composite, and (3) mixed failures, when fracture lines occur between composite and dentin as well as inside the dentin. The apical and cervical diameters of the filling were measured using a multifocus microscope (Keyence), and the bonding surface was calculated as a truncated cone and intracanal bond strength was calculated in MPa.

### Statistical Analyses

The statistical analyses were performed using SPSS (Version: 28.0.1.0), significance level was put to 0.05. Non-parametric tests were used; generalized estimating equations were used to analyze the results of the push-out test and Chi-square test to analyze the failure modes.

## RESULTS

**Fig 4:**
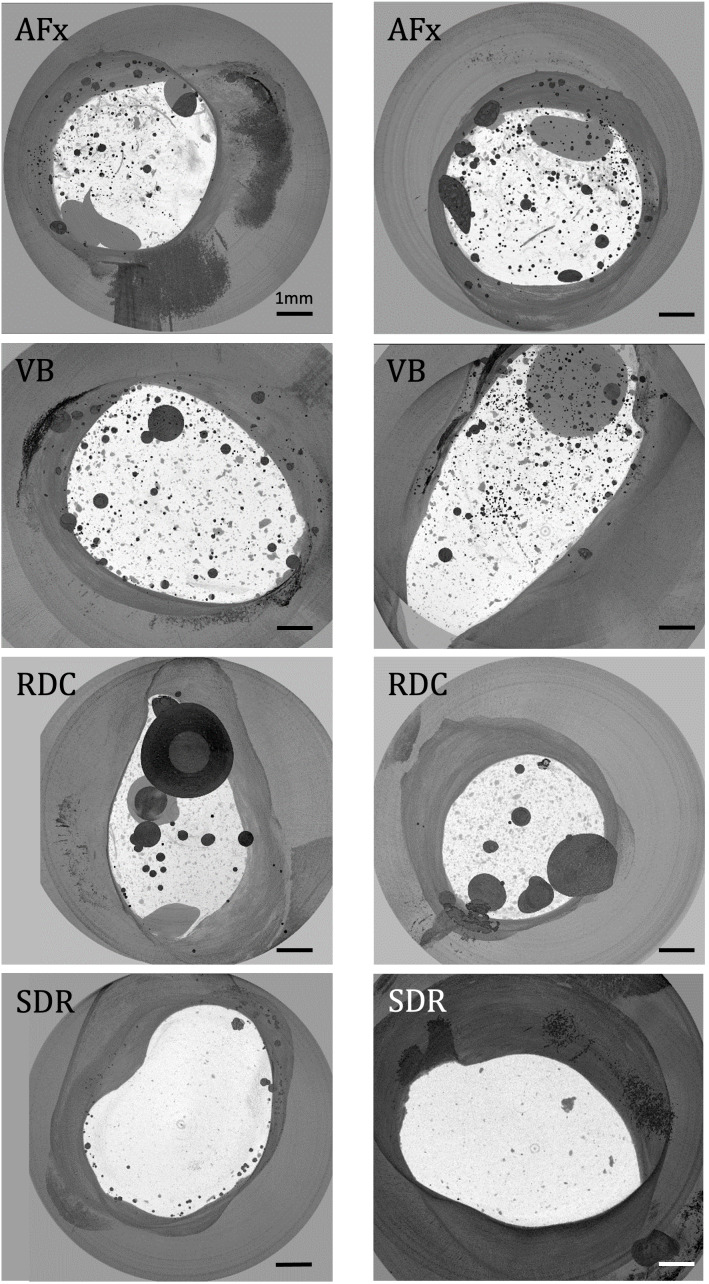
Minimum intensity projections of the µCT scanned samples. The projections show an overview of the composite filling inside the root canal from the coronal. All materials are shown with a minimum gray value for the three-dimensional region of interest projected on one slide. Air is thus shown in dark gray. AFx shows fillings made of high-viscous composite (Admira Fusion-xtra; VOCO), VB fillings made of the heat modulated composite (VisCalor bulk; VOCO), RDC are made of dual curing Rebilda DC (VOCO), SDR shows fillings made of the low viscous SDR (Dentsply). The projection shows a correlation between the size, shape, and frequency of voids and the viscosity of the composite used. Most voids are visible in the high-viscous composites Admira Fusion-xtra, and VisCalor bulk (VOCO), Rebilda DC (VOCO) includes fewer voids but of greater volume, and SDR (Dentsply) shows the least voids of small size and placed in the periphery.

PCE-µCT scans of unloaded samples clearly revealed the interfaces between dentin, adhesive and filling materials (Fig 3) and allowed differentiation between the various phases. The adhesive layer showed a varying thickness along the filling space in all samples (# Fig 3). Inside the composite itself, filler particles, entrapped air, and contact lines between the different composite increments are visible (* and arrows in Fig 3). However, the virtual sections of the µCT suggest tight contact between the filling increments. Minimum intensity projections show entrapped air frequently in all materials. However, with increasing viscosity of the composite, a higher number of voids was detected (AFx > VB > SDR > RDC; Fig 4, Table 2, first column). The core build-up material showed only a small number of voids, but which were big in size (Table 2, columns two and three). On the contrary, the flowable composite demonstrated only a few small, entrapped pores, mainly at the outer periphery of the material next to the root canal dentin (Table 2, column four). Additionally, it was noticeable that VB and RDC demonstrated big voids at the bottom of the cavity that neighbored the root-filling material gutta-percha, while AFx shows fewer voids in this area and SDR none. Intracanal bond strength was significantly affected by the intracanal filling material (P = 0.001; generalized estimating equations) and the location (Table 3), with higher values for the coronal slices closer to the pulp chamber and decreased bond strength deeper inside the root canal (P < 0.005; generalized estimating equations). VB (13.5MPa ± 5.3MPa) showed significantly lower bond strength compared to RDC (19.6MPa ± 7.6MPa) and AFx (21.4MPa ±  7.6MPa) but did not differ significantly from SDR (20MPa ±  10.3MPa) (Fig 5). All groups demonstrated predominantly adhesive failures (Fig 6a) between composite and dentin (P < 0.05; Chi-square test, Table 4).

**Table 2:** Detailed analyses of the µCT scans allow to the evaluation of the following material characteristics

Sample	Number of pores	Pore volume in mm^3^	Porosity in %	Mean distance between pore and root canal dentin in µm ± SD
AFx 1	332	0.0026	0.052	148.2 ± 201.9
AFx 2	296	0.0093	0.269	163.8 ± 105.7
VB 1	244	0.0116	0.223	222.3 ± 129.4
VB 2	256	0.0091	0.116	228.1 ± 157.9
RDC1	87	0.3495	6.894	293.2 ± 123.5
RDC2	42	0.0135	0.364	179.7 ± 127.1
SDR 1	136	0.0014	0.025	55.4 ± 42.7
SDR 2	55	0.0003	0.005	63.9 ± 108.5


**Table 3:** The mean and standard deviation of the push-out bond strength are given per group for the different cutting planes, as well as the pooled push-out bond strength used for statistical analyses

Group	Slice	Number ofSlices	Mean ± standard deviation (MPa)	Pooled mean ± standard deviation (MPa)
AFx	Coronal	10	24.3 ± 3.9	21.4 ± 7.6
Middle	8	17.7 ± 9.6
apical	2	8.1 ± 3.9	–
VB	Coronal	9	16.3 ± 4.6	13.5 ± 5.3
Middle	8	10.3 ± 4.4
Apical	2	3.2 ± 3.1	–
RDC	Coronal	10	21.7 ± 7.4	19.6 ± 7.6
Middle	8	17.1 ± 7.6
Apical	3	11.5 ± 2.6	–
SDR	Coronal	10	24.4 ± 9.8	20 ± 10.3
Middle	6	12.7 ± 6.3
Apical	1	16.9 ± –	–
				

**Table 4:** Results of failure modes during push-out bond strength testing

Group	Failure	n
Adhesive	cohesive inside dentin	Mixed
AFx	16	0	4	20
VB	18	0	1	19
RDC	16	1	4	21
SDR	16	0	1	17
	86%	1%	13%	100%


## DISCUSSION

In the present study the preheated bulk-fill composite (VB) showed significantly lower push-out bond strength compared to the high-viscous bulk-fill ormocer (AFx) and the low viscous dual-curing build-up composite (RDC); consequently, the hypothesis of the present study, that the composite itself does not influence the push-out bond strength has to be rejected.

Due to the lack of studies focusing on intracanal composite fillings in mature teeth, the results of this study were compared to studies that tested primary teeth, although the effects of tooth structure variations on adhesion cannot be excluded. Primary and mature dentin exhibit differences that need to be considered when comparing the mechanical behavior. Lower concentrations of calcium and phosphate and a higher number of dentin tubules in primary dentin,^43^ make the substrate more prone to etching, resulting in reduced microtensile bond strengths.^63^ Mosharrafian and Sharifi tested intracanal composite fillings with a length of 3 mm in primary teeth and demonstrated no significant differences for push-out bond strength between bulk-fill and conventional composites.^38^ Another study tested the effects of different bonding agents and adhesive strategies on the push-out bond strength of a resin-based composite anchorage in primary teeth and concluded that the use of different bonding strategies does not significantly affect the push-out bond strength.^6^ These data have also been confirmed in permanent teeth: bond strengths of universal adhesives did not differ between an etch-and-rinse or self-etch mode in the cervical region of the root canal.^18^ In summary, the adhesive strategy for universal adhesives in the cervical region of the root canal seems not to affect bond strength. Consequently, the etch-and-rinse approach in the present study seems to be suitable for a universal adhesive. For all groups in the present study the push-out bond strength decreased with increasing depth of the filling inside the root canal. These results corroborate with earlier studies comparing the bond strength of composites bonded to root dentin in varying depths.^32^ An explanation for this finding could be the reduction of dentin tubuli density per mm^2^ from coronal to apical, shown by Harrán Ponce et al^20^ and Wakabayashi et al.^68^ Both studies showed that the number of dentinal tubuli openings decreased from the coronal pulp chamber wall to the middle third of the root. This reduction of dentinal tubuli may have a negative effect on the shear bond strengths of different bonding systems when pulp floor dentin is compared to more coronal regions (pulp horn, middle of the pulp chamber).^2,8^ Furthermore, a recent review by Van Ende et al^65^ questioned adequate polymerization of bulk-fill materials applied in 4 mm thick increments. Lower rates of conversion may affect push-out bond strength in the deeper areas of the cavities.

Bacterial leakage in vivo due to inadequate sealing of the root canal filling may lead to failure of endodontic treatment.^12^ Tight sealing of the root canal filling is therefore mandatory to prevent reinfection of the root-canal-treated tooth.^41^ Consequently, tight adaptation and adhesion of restoration materials to root-canal-filling materials and root canal dentin are supposed to have a positive impact on the outcome of root-canal-treated teeth. A recent meta-analysis of *in-vitro* studies about filling materials in contact with dentin and gutta-percha, including glass ionomer cement (GIC), resin-based composite, or mineral trioxide aggregate (MTA) concluded that a filling in contact with the gutta-percha regardless of the material used significantly reduces microleakage.^16^

Additionally, the application of filling materials within the root canal orifice has been demonstrated to enhance the fracture resistance of teeth that have undergone *in-vitro* restoration utilizing a range of filling materials.^1,11,19,45^ The efficacy of calcium silicate cements, GIC, resin-modified GIC, and diverse resin-based composites has been evaluated in comparison to teeth that have not undergone filling procedures. In all instances, the incorporation of a filling material has been observed to enhance the fracture resistance of the samples. However, the aforementioned studies have yielded conflicting results with regard to the material that has been identified as the primary contributor to enhanced fracture resistance. Two studies concluded that different composite materials increased fracture resistance compared to MTA, calcium silicate cement, GIC, resin-modified GIC, and bonded amalgam,^1,45^ but one study showed higher fracture resistance of resin-modified GIC compared to MTA, fiber-reinforced composite, or nanohybrid composite.^19^ On the contrary, another study revealed the highest fracture resistance for a calcium-silicate-based cement (Biodentine) in comparison to GIC, resin-modified GIC, or nanohybrid composite.^11^

Also, a 12-month clinical study addressed this topic and controlled if the material in contact with the gutta-percha induced differences in apical healing; a combination of GIC and composite to composite alone was tested. The authors could not demonstrate a significant effect of the material on the outcome of the endodontic treatment.^27^

In the present study, the teeth had great class IV defects that were restored using a direct composite filling. This approach is in line with the results of a meta-analysis of clinical trials revealing survival rates of 90% after 10 years for anterior teeth restored using direct composite fillings in class IV defects.^22^ Additionally a systematic review of clinical trials also supported the use of composites for the definitive restoration of root-canal-treated teeth.^55^

Rejecting an intracanal post in the present study when restoring endodontically treated teeth is a valid option for restoring teeth with class IV defects, even though one sample failed during artificial aging. Because comparable results were found even for teeth with no residual coronal tooth structure after restoration with intracanal composite and full covering crowns.^25,59^ Also, the combination of composite fillings and glass fiber posts is not supported by the literature since these restorations did not show a positive effect compared to composite alone, when restoring maxillary front teeth with class III defects.^67^ Consequently, intracanal composite fillings can seal the root-canal-filling material to prevent bacterial leakage and function as an ICA to increase the bonding surface to the dentin for coronal restoration without placing a classic post and core restoration.

PCE-Ct scans are an established method for imaging neighboring materials with similar radiopacities, as the optimization of the contrast enables differentiation.^36,57,58^

However, there are still issues, particularly when quantifying findings like air inclusions or materials without or low radiopacity like dental adhesives.^62^ Semi-automated analysis, as used in this study, can lead to over- or under-segmentation of different material phases, especially when the samples differ in their overall shape between each other and thus resulting in different radiographic transmission and thus different image quality. Manual analysis by an investigator would be a time-consuming alternative, and subject to individual perception.

In the present study, composites of high viscosity (AFx and VB) (Fig 4) showed more voids inside the composite material compared to the flowable SDR (Fig 4) and the core build-up material RDC (Fig 5), in turn RDC shows the largest voids. Voids inside the high-viscous composites, AFx and VB, were visible all over the filling area, whereas voids inside the low viscous composites SDR and RDC were especially located in the periphery close to dentin or gutta-percha (Fig 4).

Nevertheless, based on the bond strength data of the present study it can be assumed that the increased number of small voids inside the high-viscous composites does not have a significant effect on the push-out bond strength, since AFx and VB with opposing push-out bond strengths showed most voids. These findings have been corroborated in other micro-CT controlled studies on adhesively luted intracanal posts where entrapped air due to voids inside the luting material did not seem to affect push-out bond strength.^47,64^ It remains uncertain whether clinical consequences can be derived from this, irrespective of whether the gaps in question are between interfaces or trapped voids. Since controlling these factors in clinical scenarios seems difficult, interfacial gaps between composite and dentin can expand during artificial aging, especially in the region of the gingival margin, as shown in a µCT controlled study.^54^ But even if this gap growth is clinically relevant cannot be answered clearly, as no correlation was identified between secondary caries formation and the gap width between composite and dentin in a study that used samples placed in an occlusal splint worn by 14 volunteers for three weeks.^28^ The situation is similar with the entrapped voids, they can change their outer shape during artificial aging,^59^ but again, it is not yet possible to derive any clinical consequences from this.

An explanation for the differences in void formation between the materials could be given by the application mode. High-viscous materials needed condensation into the cavity, thereby air could be mixed into the material. The core build-up material that comes in an automix apparatus revealed a special void formation, which could be caused by the mixing of the two components inside the mixing tip as shown in earlier studies.^49^ The flowable composite SDR, which showed minimum void formation, was just placed inside the cavity without further manipulation. Consequently, the application mode does affect the occurrence of voids.^4^ Additionally a study by Nilsen et al^42^ stated that inside unopened composite compules that were scanned using µCT pores inside all materials are visible. Additionally, the study scanned various composites inside a class I cavity in first molars and showed that with a flowable bulk-fill material (SDR), less pores occurred compared with high-viscous composites,^42^ which is comparable to the present study.

The PCE-CT scans in the present study showed an adhesive layer of varying thickness (Fig 3). However, it is unlikely to achieve an adhesive layer with uniform thickness inside the root canal since the anatomical conditions (ie, irregularities and isthmuses) hamper adhesive application. In addition, removing the excess adhesive inside the root canal using paper points is more difficult and less controllable than in the crown of the tooth where the adhesive can be evenly distributed using air draft. A thicker adhesive layer can reduce the bond strength of composite restorations to human dentin as already shown in former studies.^15,35^ Nevertheless, this factor did not seem to result in differences in push-out bond strength in the present study, since a non-uniform adhesive layer was observed for all groups. The question if the inhomogeneous adhesive layer affects the push-out bond strength cannot be answered with the present data, but it seems that the application procedure used inside the root canal cannot guarantee a uniform adhesive layer. The predominant adhesive failure between dentin and composite during push-out testing (Fig 6a), shows that despite the entrapped voids inside the composites, the materials seem stable enough to resist the acting forces.

It can be concluded that the survival rates after thermomechanical loading and the push-out bond strength values indicate a sufficient bonding of all ICA materials. The PCE-CT scans indicate material-specific void formation and varying adhesive layer thicknesses independent of the material. Nevertheless, the question of if and how internal void formation inside composites affects stress formation during loading and subsequently push-out bond strength still needs to be addressed in future research. Therefore, further studies that evaluate the effects of entrapped air on strain and stress distribution during composite polymerization and subsequent loading are required.

### Acknowledgment

The authors kindly thank Timm Weitkamp (Anatomix Beamline, Synchrotron SOLEIL – L’Orme des Merisiers – Départementale 128, 91190 Saint-Aubin, France) for support with the micro-CT scans and Dr. Konrad Neumann (Institute of Biometry and Clinical Epidemiology, Charité – Universitätsmedizin Berlin, Germany) for support during the statistical processing.

### Clinical Relevance

This *in-vitro* study indicates the possible use of different resin composite material classes (bulk-fill composites, dual-curing core build-up material, and bulk-fill ormocer) as ICA for direct coronal restorations. Direct class IV composite fillings in anterior teeth appeared to be a reliable and hard-tissue-saving option *in vitro*. Void formation inside the composite bulk were frequently observed and highlight the challenge of composite application even inside the cervical part of the root canal.

## REFERENCES

**Fig 1a to e:** Workflow of sample preparation and data acquisition. (a) Prepared class IV cavity, the bevel of 1 mm is marked in red and root canal treatment is performed. (b)3D reconstruction of the restored tooth after root canal treatment (RCT) and composite filling. Enamel is set to semi-opaque, and dentin’s translucency was increased (blue) to visualize the coronal filling and ICA material (ocher), gutta-percha (green), and sealer (red). (c) The virtual longitudinal µCT slice reveals material homogeneity. The red lines indicate where 1 mm thick slices were extracted for push-out bond strength testing. (d) Push-out bond strength testing on 1 mm thin slices using a universal testing machine. (e) Analyses of fracture patterns using an optical multifocus microscope.

**Fig 3a and b:** 2D virtual longitudinal slices obtained by PCE-CT showing composite fillings inside the root canal. Adhesive layers of different thicknesses (#) are visible in both samples. Entrapped voids are marked with asterisks (*) and the interface between different increments with an arrow, as well as remnants of sealer (×). (a) Rebilda DC (VOCO), high viscous; (b) Admira Fusion-xtra (VOCO), low viscous.

**Fig 5a and b:** Boxplot of the push-out bond strength in MPa per group and sample slice. Values of the coronal (first) slice are shown in light blue, and values for the second (middle) slice are in dark blue. When comparing the pooled push-out testing values (burgundy) of all slices per group, VB (a and b) showed significantly lower values compared to AFx (a) and RDC (b) (P = 0.001). Groups that show significant differences from each other are marked with the same super-indicated letter.

**Fig 6a and b:** Images of samples after push-out bond strength testing. Both pictures show an intracanal composite filling surrounded by dentin. Fracture lines are marked with arrows. (a) Adhesive failure between composite and dentin (black arrows). (b) Mixed failure with fractures between composite and dentin (black arrows), but also inside the dentin (blue arrows).








